# Tensile Experiments and Numerical Analysis of Textile-Reinforced Lightweight Engineered Cementitious Composites

**DOI:** 10.3390/ma15165494

**Published:** 2022-08-10

**Authors:** Mingzhao Chen, Xudong Deng, Rongxin Guo, Chaoshu Fu, Jiuchang Zhang

**Affiliations:** 1 Yunnan Key Laboratory of Disaster Reduction in Civil Engineering, Faculty of Civil Engineering and Mechanics, Kunming University of Science and Technology, Kunming 650500, China; 2 Yunnan Highway Science and Technology Research Institute, Kunming 650051, China; 3 Department of Civil Engineering, Yunnan Minzu University, Kunming 650504, China

**Keywords:** fiber-reinforced polymer, lightweight engineered cementitious composites, numerical analysis, textile grid, repair and reinforcement

## Abstract

Despite many cases of textile-reinforced engineered cementitious composites (TR-ECCs) for repairing and strengthening concrete structures in the literature, research on lightweight engineered cementitious composites (LECC) combined with large rupture strain (LRS) textile and the effect of textile arrangement on tensile properties is still lacking. Therefore, this paper develops textile-reinforced lightweight engineered cementitious composites (TR-LECCs) with high strain characteristics through reinforcement ratio, arrangement form, and textile type. The study revealed that, by combining an LRS polypropylene (PP) textile and LECC, TR-LECCs with an ultimate strain of more than 8.0% (3–4 times that of traditional TR-ECCs) could be developed, and the PP textile’s utilization rate seemed insensitive to the enhancement rate. The basalt fiber-reinforced polymer (BFRP) textile without epoxy resin coating had no noticeable reinforcement effect because of bond slip; in contrast, the BFRP grid with epoxy resin coating had an apparent improvement in bond performance with the matrix and a better reinforcement effect. The finite element method (FEM) verified that a concentrated arrangement increased the stress concentration in the TR-LECC, as well as the stress value. In contrast, a multilayer arrangement enabled uniform distribution of the stress value and revealed that the weft yarn could help the warp yarn to bear additional tensile loads.

## 1. Introduction

Fiber-reinforced polymer (FRP) is extensively applied in the repair and strengthening of reinforced concrete structures [1,2,3,4] following numerous theoretical designs on the strength of FRP materials [5,6,7,8], fiber-reinforced concrete [9], and slope and foundation reinforcement [10,11]. Its advantages include light weight, high strength, corrosion resistance, and durability [12,13]. Epoxy resin is generally employed as the binder when FRP is used to strengthen structures since it works well in concert with existing structures. However, epoxy resin still has flaws, including easy aging [14], low adaptability [15], brittle damage causing interface peeling [16], and the emission of irritating poisonous gases, which significantly reduces the effectiveness of FRP reinforcement [17]. Therefore, the combination of epoxy resin and FRP cannot fully exert the effect of strengthening the structure.

To address the shortcomings of organic resins, efforts have been undertaken to substitute organic binders such as epoxy resins with inorganic bonding materials, e.g., cement-based matrices. The high-performance ferrocement laminate (HPFL) [18] and the textile-reinforced mortar/concrete (TRM/TRC) approach have also been proposed [19]. TRM is a cementitious composite material composed of a fine-grained mortar matrix reinforced by textiles. It has high tensile strength, as well as multiple cracking behaviors under loading, and it can reduce the crack width [20]. It is typically used for flexural [21] and shear reinforcement of concrete [22], as well as repair reinforcement of concrete columns and thin shell structures [23,24]. Although the crack width can remain small, the multiple cracking stages of TRM structures are typically brief, lasting only until roughly 0.5% of the tensile strain [25]. The textile near the crack is vulnerable to debonding from the matrix section due to stress concentration, and this is also when the matrix at the cracking spot can no longer transmit loads. As a result, the reinforcing system fails before the FRP reaches the maximum damage state [26,27]. Although TRM and HPFL can improve the mechanical properties of concrete components, inorganic bonding materials still suffer from interfacial peeling, poor compatibility with FRP materials, low elongation, and brittleness.

In recent years, high-ductility engineered cementitious composites (ECCs) have become an alternative matrix because the tensile properties of the ECC materials and the bridging ability of short fibers to the matrix compensate for the brittleness and interfacial peeling problems of cementitious materials. ECCs are inorganic cementitious materials reinforced by short fibers with ultimate strains of more than 3%, multiple cracking, and reasonable ductility and toughness [28]. The crack width in ECCs can be less than 100 um during the strain-hardening stage [29]. Therefore, ECC materials have higher ductility and durability compared with TRM. Yang et al. [30] and Zheng et al. [31] used FRP-reinforced ECCs for flexural strengthening of RC beams. They found that the resulting TR-ECC system was very effective because the ECC’s strain-hardening and multiple cracking behaviors alleviated the stress concentration at the interface between TR-ECCs and concrete, thereby suppressing interfacial peel failure. According to Chen’s [32] study, BFRP textile-reinforced ECC-constrained columns outperformed TRM-deprived columns in terms of ultimate load capacity. The ECC’s tensile properties and the textile’s outstanding bonding with the ECCs significantly delayed the onset of cracks and maintained structural integrity. Numerous studies have demonstrated the many benefits of TR-ECCs for structural reinforcement. It has been confirmed that textiles can rely on the bridging action in the ECC matrix to transfer loads and maintain good bonding properties because of the strain-hardening ability, multi-cracking behavior, and ultrahigh-ductility properties of the ECC materials [33]. TR-ECCs can repair reinforcement protection for building systems [31] and engineering applications for structural seismic energy resistance [34]. Compared with TRM, TR-ECC can not only prevent interfacial peeling damage but also improve the use efficiency of textiles. Therefore, ECC can replace cement mortar as a potential inorganic bonding material.

There was no relative slip at the interface between the BFRP grid and the ECC matrix when Zheng et al. [35] applied BFRP-reinforced ECC, according to numerous investigations on the tensile mechanical properties of TR-ECCs. At different BFRP grid reinforcement rates, the load-carrying capacity of TR-ECCs specimens rose from 42% to 172%. Li et al. [36] studied the impact of textile volumetric ratios, textile geometry, and matrix thicknesses on tensile mechanical characteristics and demonstrated that TR-ECC composites could enhance textile reinforcing to a significantly greater extent than TRM materials. The textile spacing impacts how the matrix is impregnated onto the textile, which impacts the ability of the matrix and textile to link together. In contrast, the matrix thickness impacts the bridging mechanism of short fibers. Zhang et al. [37] demonstrated that the volume proportion of short fibers and the number of textile layers had a dominant influence on the tensile behavior of TR-ECCs by examining the matrix type, volume fraction of short fibers, and number of textile layers. The matrix type, however, had a negligible effect. The textile also demonstrated multi-seam cracking, strain hardening, and good crack width management, greatly increasing its tensile qualities. Obviously, the study of the tensile mechanical properties of TR-ECC has demonstrated the superiority of ECC as an inorganic binder material.

These investigations often used materials with high modulus and low elongation (typically less than 3%), such as BFRP and carbon fiber-reinforced polymer (CFRP), and they produced TR-ECC tensile properties less than 2%. However, the tensile properties of ECCs are typically larger than 3%, especially recently created ECCs with 8% tensile strain [36]. The high-ductility properties of ECCs are not entirely utilized when using traditional small-strain BFRP, and CFRP is reinforced with large-strain ECCs. For some buildings with high seismic requirements, LRS-FRP shows a competitive advantage [38,39] because various components of concrete restrained with LRS-FRP show a great energy dissipation capacity [40,41]. Therefore, it is necessary to study the tensile mechanical properties of the LRS textile combined with the ECC matrix.

To increase the tensile deformation capacity of TR-ECC, it is, therefore, essential to produce reinforced materials with LRS; however, there are relatively few reports on this aspect. Additionally, ECCs with lightweight characteristics have recently been created with densities of 1300–650 kg/m^3^. In large-span, weight-sensitive buildings, these ECCs have demonstrated more significant advantages; nevertheless, the usage of reinforced material in conjunction with these ECCs has not been reported. Therefore, we take the combination of LECC and LRS materials in the paper and investigate the effects of textile type, reinforcement rate, and arrangement form on the tensile properties of TR-LECC, aiming to explore the feasibility of developing TR-LECC with LRS materials and LECC. Overall, we found that the combination of LRS textiles and LECC could develop TR-LECC with similar strength to conventional TR-ECC but 8.0% ultimate strain (3–4 times that of conventional TR-ECC).

## 2. Test Program

### 2.1. Performance of Materials

Polypropylene (PP) is a thermoplastic resin made from the polymerization of propylene, which has good resistance to acid, alkali, corrosion, and aging, as well as high tensile strength, lightweight properties, high ductility, and low cost. The ductility of PP textile can reach 10% when strength is considered [42], and the tensile stress of PP textile increases with strain under tensile stress. Therefore, combining a high-ductility LECC matrix and LRS-FRP can not only bring out the reinforcing properties but can also give full play to the ductility properties of the LECC matrix.

**Figure 1:**
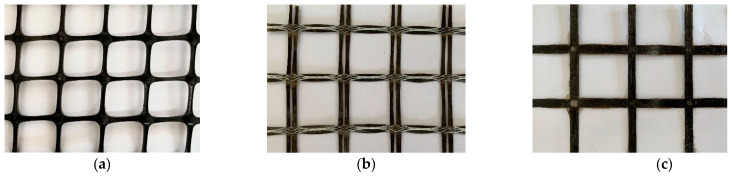
The specific shape of the textile (**a**) PP, (**b**) BFRP-F, (**c**) BFRP-T.

Continuous basalt fiber is a new type of high-performance inorganic material, with the advantages of good stability, corrosion resistance, anti-combustion, and high-temperature resistance. The raw material is natural, environmentally friendly, and low-cost. Continuous basalt fiber is divided into two forms: equally spaced dry fiber without epoxy resin impregnation, called BFRP textile (BFRP-F), and epoxy resin-impregnated basalt textile forming a BFRP grid (BFRP-T) after the epoxy resin is cured. Detailed information on the three textile types is shown in Figure 1 and Table 1.

### 2.2. Design of the Experiment

To investigate the tensile properties of the LECC matrix according to textile type, enhancement rate, and arrangement form, a total of 11 groups of tests were designed; each group contained three identical specimens, with a total of 33 axial tensile specimens, including five groups of PP textile-reinforced LECC composite specimens, three groups of BFRP textile-reinforced LECC composite specimens, and three groups of BFRP grid-reinforced LECC composite specimens. The detailed test parameters are shown in Table 2. The length of the TR-LECC specimens was 200 mm, the width was 70 mm, and the casting thickness was 15 mm.

### 2.3. TR-LECC Specimen Preparation

**Figure 2:**
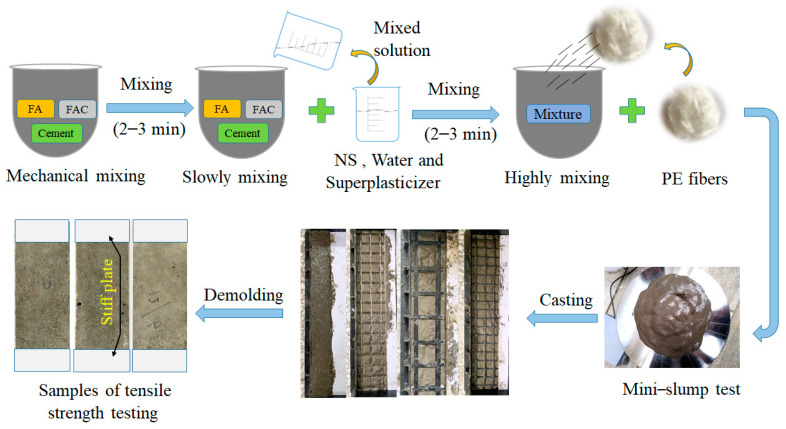
Flowchart of specimen preparation. Note: FA, fly ash; FAC, fly ash cenospheres; NS, nano-silica.

Ordinary silicate P.O.52.5 grade cement was used as the main cementitious material, and F-type fly ash (FA) was used as the supplementary cementitious material. Fly ash cenospheres (FACs) used as fine aggregate are waste collected in coal-fired power plants with a diameter of 0.01–0.5 mm and a density of 530 kg/m^3^. The addition of nano-silica with a particle size of 40 nm improved the mechanical properties of LECC. The LECC matrix was stirred by a mixing pot, and we adopted a layered casting process [36,37], as shown in Figure 2. The steps for making standard specimens were as follows: (1) pour a layer of the LECC matrix, and then vibrate the LECC on the vibrating table; (2) flatten the matrix, and paste the cut reinforcement textile on the LECC matrix; (3) fix the position of the reinforcement textile, and continue to pour the next layer of the LECC; (4) cover the surface of the specimen with a film to prevent moisture evaporation after the vibrating process is finished. After the cast specimens were cured at room temperature for 24 h, the specimens in the mold were removed and maintained at a temperature of 20 ± 2 °C and 95% relative humidity for 27 days.

### 2.4. FRP and TR-LECC Tensile Test

**Figure 3:**
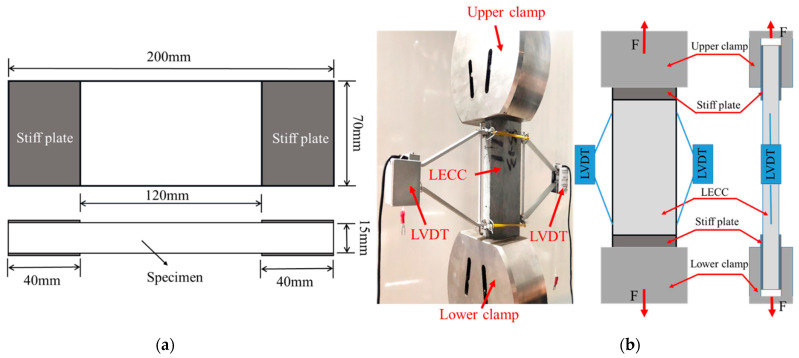
(**a**) Dimensions of the TR-LECC specimen and (**b**) test setup.

The load was controlled at a constant displacement of 0.5 mm/min under a static tensile load. The testing machine executed the test procedure, and the test was terminated after the specimen was damaged [36]. The specimen strains were collected with a linear variable differential transformer (LVDT) of a 100 mm scale length, and the ends of the gauge were fixed in the test length interval, as shown in Figure 3. All the tests were carried out to ensure that the damage occurred in the middle position (within the measured length of the specimen) away from the aluminum sheet, and accurate tensile test results were obtained.

## 3. Results and Discussion

### 3.1. Matrix Material

**Figure 4:**
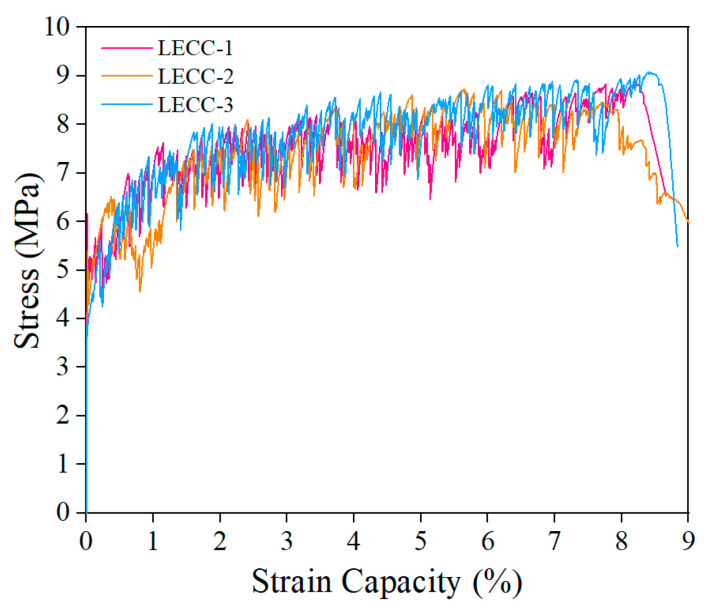
Stress–strain curve of the LECC.

The design of LECCs as a bonded matrix was based on the study of Fu et al. [43,44]. The uniaxial tensile behavior of the matrix was tested using “dog-bone” specimens according to the recommendation of JSCE [45], and the obtained tensile stress–strain curves are shown in Figure 4. The specific mechanical properties and material mix ratios are shown in Table 3 and Table 4, respectively.

### 3.2. Tensile Behavior of Reinforced Materials

**Figure 5:**
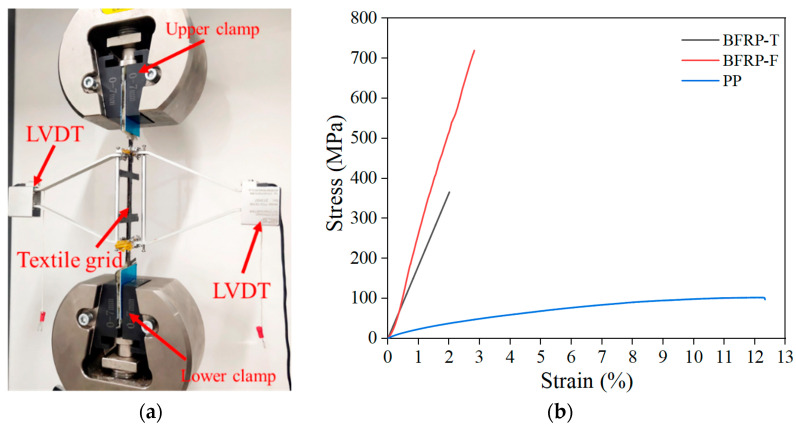
(**a**) Test setup; (**b**) stress–strain curves of different materials.

The PP textile used in the test was provided by Shandong Lianshun Engineering Materials Co. BFRP textile and BFRP grid materials were provided by Shandong Dalu Engineering Materials Co and Jiangsu Green Material Valley New Material Technology Development Company, respectively. The key mechanical properties of the reinforcement textile were measured in the laboratory, as shown in Table 5. The mechanical properties of the reinforced textile were obtained by tensile test, as shown in Figure 5a. The tensile test was carried out on a 300 KN MTS testing machine, and a 200 mm length single yarn sample was tested with a displacement loading method of 0.5 mm/min.

The stress–strain curve of the PP textile during the entire tensile stage was nonlinear, and the growth rate of tensile stress decreased gradually with the increase in strain. In the tensile test, the PP textile showed a phenomenon similar to the “necking” of steel bars, where the stress and strain continuously increased. Finally, the test ended with a sudden fracture at the nodes of the PP textile, during which the PP textile showed excellent ductility performance, as shown in Figure 5b.

The uniaxial tensile tests yielded the primary failure modes of two different structures of basaltic materials: (1) the BFRP textile not impregnated with epoxy resin had an apparent nonlinear behavior observed at the initial stage of loading, and the nonlinear behavior gradually changed to a linear behavior when a specific load was reached, with a gradual loss of load-bearing capacity as the number of fractured slender filaments increased; (2) the stress–strain curves of the epoxy resin-impregnated BFRP grids were linearly correlated, and the damage behavior was that of an abrupt fracture. The Young’s modulus was obtained by extracting the area of the linear section of the BFRP textile and the BFRP grid; the maximum stress in the linear section was used as the fracture strength, and the maximum strain was used as the fracture strain. The stress–strain curves of the three materials are shown in Figure 5b.

### 3.3. Tensile Stress–Strain Curve of TR-LECC

**Figure 6:**
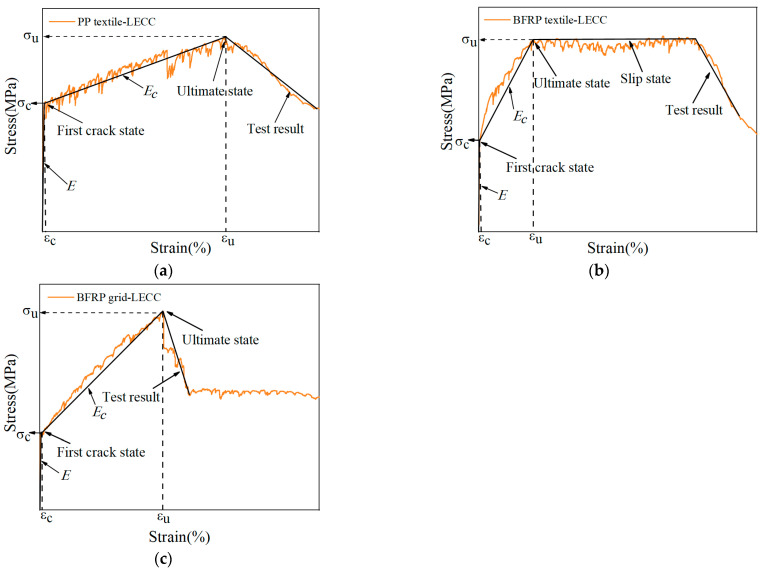
Stress–strain curve of reinforced textile type: (**a**) PP textile, (**b**) BFRP textile, and (**c**) BFRP grid.

The experimental results showed that two different stress–strain curves characterized the tensile behavior of the TR-LECC. The PP textile and the BFRP grid-reinforced LECC matrix had a two-stage characteristic curve. In contrast, the BFRP textile had a three-stage characteristic curve, and similar characteristics have been observed in the literature [35], as shown in Figure 6. The first two stages were characterized similarly for all three materials. The first stage was the behavior of linear elasticity, where stress and strain were linearly related until the first crack appeared in the matrix. The second stage was strain-hardening behavior following matrix cracking. The matrix exhibited the characteristics of multi-slit cracking, and the stage ended when the increase in the stresses reached its ultimate value. The stiffness of the composite material contributed less to this stage due to matrix cracking; hence, the stiffness of this stage decreased significantly, a phenomenon that was also observed in [46]. A comparison with the stress–strain curve of the LECC matrix showed that the fluctuation was minor because the reinforcing material could contribute to the tensile strength in the matrix, and a larger material tensile strength resulted in a smaller fluctuation. This indicates that the reinforcing material not only improved the strength of the matrix but also had a suppressive effect on the number and width of cracks in the matrix [47]. After this, the BFRP textile-reinforced LECC matrix entered the third stage, i.e., the bonding slip process of the BFRP textile in the matrix. Due to the unimpregnated epoxy resin, the bonding ability between the BFRP textile and the LECC matrix decreased; thus, the BFRP textile appeared to debond after the peak bonding strength. The tensile stress was shared by the bridging force of the polyethylene (PE) fibers and the frictional force between the BFRP textile and the matrix. Finally, the damage of the TR-LECC ended with fracture or debonding failure of the reinforcement and PE fibers that could not continue to bear the load.

**Figure 7:**
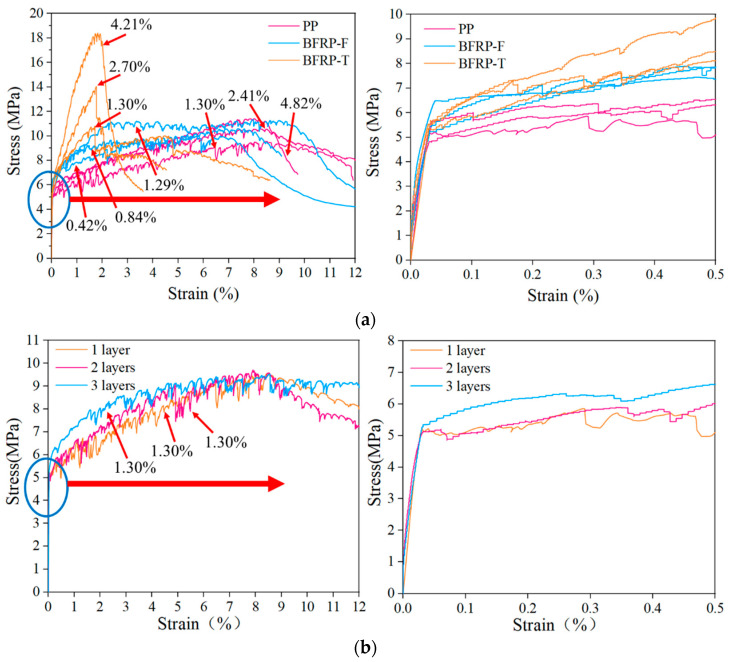
(**a**) Comparison of enhancement ratios of different reinforcement materials and (**b**) comparison of arrangement forms.

Typical stress–strain curves for the textile types at different reinforcement rates are shown in Figure 7a. Similar to the findings in the literature [30], in the elastic stage, the reinforced material did not bear additional tensile stress, and the matrix mainly bore the tensile load. Therefore, the reinforcement rate had little influence on the elastic stage, and the cracking strength of the matrix was not improved. In the multi-slit cracking stage, the slope of the curve increased with the material reinforcement rate, which indicates that the material reinforcement rate could improve the tensile stiffness of the specimen after cracking. The different textile materials had a significant effect on the slope of the curve, which was determined by the materials’ inherent characteristics; high tensile strength and good bonding performance exhibited a more significant slope of the curve. Additionally, it can be seen from the curves of the different textile materials that the increase in the enhancement rate reduced the fluctuation range of the curve, indicating that the enhancement rate had an inhibitory effect on cracks [48]. Because the tensile stress after the cracking of the matrix was mainly provided by the reinforcement material, which replaced the PE fibers in the matrix to assume the bridging role between the cracks to inhibit the further development of the cracks, this resulted in a significant reduction in the number of cracks in the specimens [49,50].

The stress–strain curves of PP textile under different arrangements are shown in Figure 7b. The difference in the arrangement form had little effect on the elastic stage of the specimen. In the strain-hardening stage, it can be seen that the increase in the number of arrangement layers improved the reinforcement effect, but there was no difference in the final ultimate stress and ultimate strain. After the failure of the specimen, the stress of the textiles arranged in multiple layers decreased gradually, and the textile arranged in one layer dropped rapidly and soon lost their bearing capacity.

### 3.4. Key Mechanical Parameters

**Figure 8:**
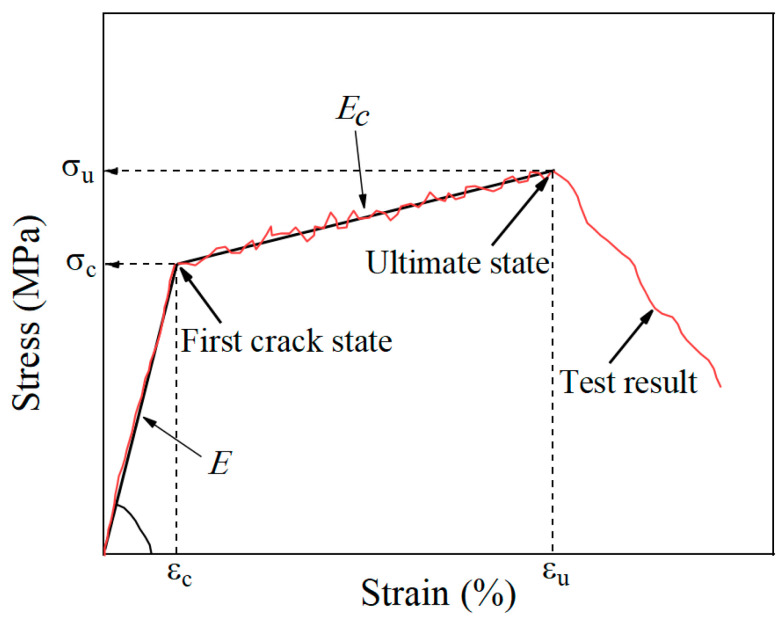
Stress–strain relationship of TR-LECC composites.

The stress of the TR-LECC was obtained as the ratio of the tensile load to the average cross-sectional area of the TR-LECC panel; the average strain was obtained by dividing the average displacement (average of displacement recorded by two LVDTs) by the length of the LVDT measurement area (100 mm) for calculation. The typical curve of the TR-LECC composite material is shown in Figure 8. The critical parameters in the curve are the cracking stress, cracking strain, and the ultimate stress and ultimate strain of the specimen at the maximum tensile force, as well as the elastic stage and multiplicity’s elastic modulus [51]. This experiment investigated the effect on critical parameters according to textile type, enhancement rate, and arrangement form.

### 3.5. Effect of Arrangement Form on LECC Matrix

**Figure 9:**
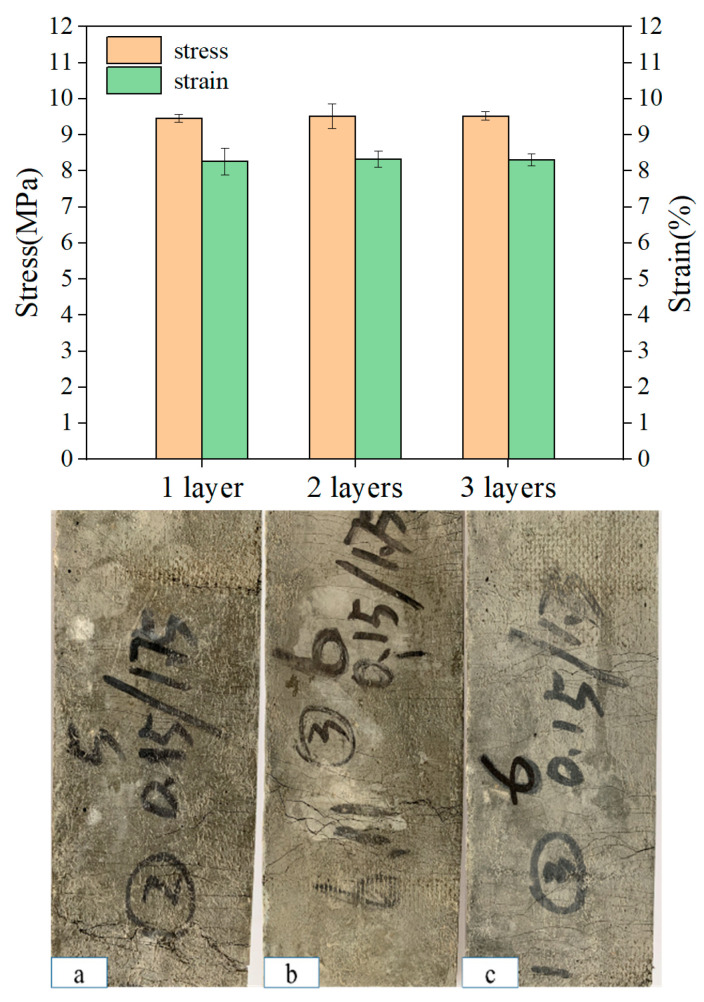
Stress–strain values and crack distribution in the tensile region with different arrangements: (**a**) one-layer textile, (**b**) two-layer textile, and (**c**) three-layer textile.

Three different layouts (one layer, two layers, and three layers) of the specimens with the same enhancement rate were evaluated, and the stress–strain values, as shown in Figure 9, were obtained from the uniaxial tensile test. The average ultimate stresses were 9.46 MPa, 9.52 MPa, and 9.53 MPa, and the average ultimate strains were 8.27%, 8.33%, and 8.31% for the one-layer, two-layer, and three-layer textile-reinforced LECC, respectively. Therefore, the arrangement form had little effect on the reinforcement and ductility properties of the LECC matrix at the same enhancement rate. Nevertheless, the change in the arrangement form affected the crack distribution and the number of cracks in the composite. Figure 9 shows the crack morphology under the uniaxial tensile tests for specimens with different arrangement forms, as also observed in [52]. The increasing number of textile layers led to apparent multiple cracking behaviors, which suppressed the development of crack width and improved the concentration of tensile stresses [53]. When one layer of the textile was arranged, the specimen cracked with increasing stress accompanied by crack generation, but the number of cracks was relatively small. When the specimen reached the ultimate tensile stress, the matrix crack was no longer generated, one of the cracks gradually widened into the main crack, and the specimen failed, as shown in Figure 9a. The matrix microcracks increased significantly with the increased layers in the arrangement. They then closed automatically after the load was removed, and the damage form of the TR-LECC changed from main crack damage to multiple crack damage, as shown in Figure 9b,c. This indicates that the formation of cracks in the one-layer arrangement is more likely to lead to stress concentration. The bridging effect of the textile in high-stress conditions is weakened, whereby it is difficult to transfer the stress to the matrix through textile; hence, the cracking of the matrix is not inhibited, the crack width is increased, and the number of cracks is reduced. However, the tensile stresses transferred between the matrix by the multilayer arrangement of the textile enhance the control of cracks such that the stresses are more uniformly distributed within the matrix, and the crack width decreases when the number of cracks increases. In addition, the problem of stress concentration in the reinforcing textile can be avoided when multiple layers of textile are arranged due to the coupling effect between the textile layers.

### 3.6. Effects of Enhanced Textile Type

**Figure 10:**
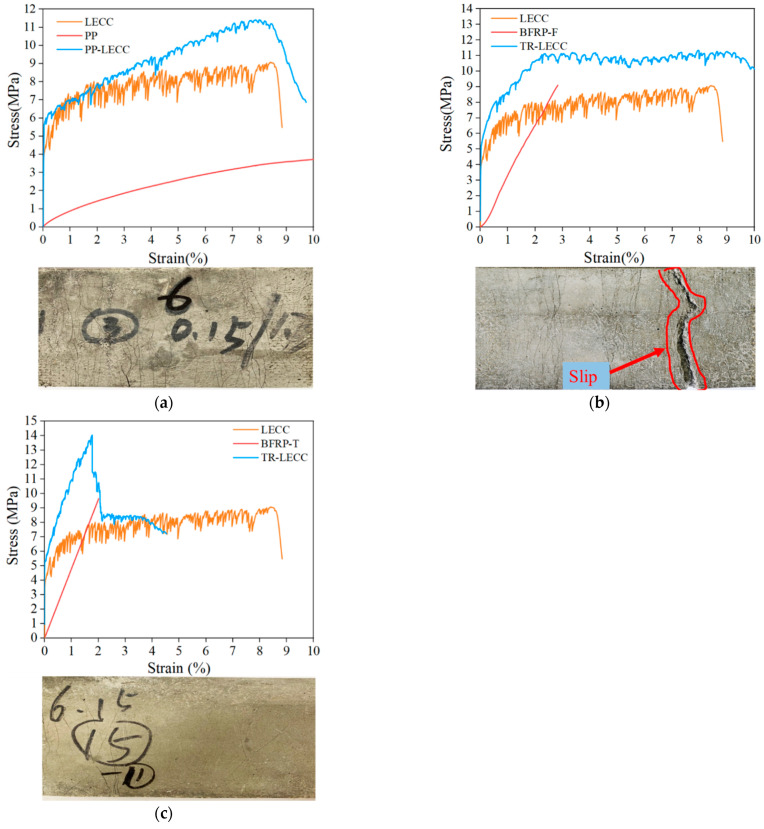
Reinforcement effect of reinforcement textile types and crack diagram: (**a**) PP textile, (**b**) BFRP textile, and (**c**) BFRP grid.

In Figure 10, the strengthening effect of the three reinforcing materials on the LECC matrix, BFRP textile, and BFRP grid shows the strengthening effect after the matrix cracking. The strengthening result of the PP textile had a lag phenomenon after the matrix cracking. The BFRP grid-reinforced LECC matrix showed the best strengthening ability; the curve’s fluctuation in the multi-slit cracking stage was slight. The sound of a BFRP grid fracture was heard when the tensile stress reaches the limit, followed by a rapid decrease in the bearing capacity of the specimen and damage. The BFRP textile was affected by the production process, and the strengthening effect was slightly weaker after the multi-slit cracking; finally, the bonding failure occurred due to a slip, and the gradual loss of friction apparently reduced the trend of the curve, with a somewhat complementary ductility performance. Because its tensile strength was not high, the PP textile could only enhance the rate of a more significant case. The BFRP grid had a comparable reinforcement effect, but the ductility performance was excellent.

Because of the differences in the materials, each reinforcing material exhibited different reinforcing effects, ductility properties, and the ability to control cracks. The epoxy-impregnated BFRP grid bonded better to the matrix interface. No significant slip between the BFRP grid and the matrix was observed throughout the tests, and this conclusion was fully verified by Dvorkin et al. [54] and Hegger [21]. However, the bonding effect of BFRP textile was mainly provided by the external basalt fiber bundle. In the case of bonding failure, it was provided by the bridging action of PE fibers and the frictional force of the inner and outer basalt fibers [51]. The PP textile had a good bonding effect and ductile deformation, and the minimum tensile strength was the reason for the insignificant strengthening effect of the PP textile. However, the combination with the LECC matrix could fully make use of the ultrahigh-ductility performance. The mechanism of action of the TR-LECC is mainly determined by the interfacial bonding properties between the fiber bundles and matrices. Good interfacial bonding properties can enable the materials to be combined and then synergistically stressed to form an excellent structure with integral properties. Therefore, the BFRP grid is more suitable as a reinforcing material but at the cost of less ductility.

**Figure 11:**
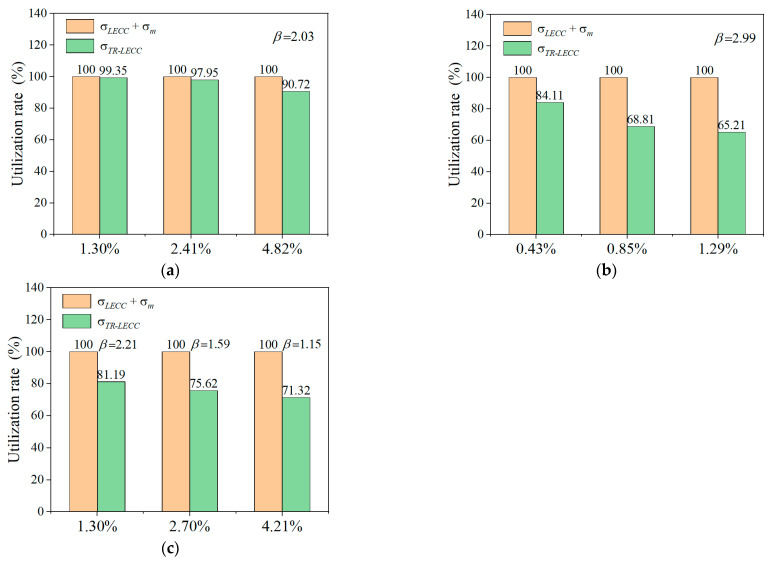
Material utilization of different textile types: (**a**) PP textile, (**b**) BFRP textile, and (**c**) BFRP grid.

From Figure 11, it can be seen that increasing the enhancement rate of different materials could increase the ultimate stress. Furthermore, the reinforcements rely on the adequate bonding of the reinforcing textile to the LECC matrix to exert its effect. The studies in [55,56] gave the average bond strength of the textile within the gelling matrix along the embedding length to compare the bonding efficiency, which is mainly related to the external perimeter of the warp and the embedding length, with the bond stress expressed as

(1) $$\tau =\frac{F}{l\cdot l_{y}},$$

where *F* is the pullout force, and *l_y_* and *l* are the warp perimeter and embedding length, respectively.

The concept of material utilization was proposed for the effect of the external perimeter and cross-sectional area of the yarn on the reinforcement effect [21]. This was expressed as the ratio of the maximum stress of the TR-LECC to the stress of the reinforcement textile and LECC.

(2) $$\Delta =\sigma_{\max,\;TR-LECC}/(\sigma_{LECC}+\sigma_{m}),$$

where $\sigma_{\max,\;TR-LECC}$ is the ultimate stress of the TR-LECC, $\sigma_{LECC}$ is the stress of the linear interpolation of the LECC matrix, and $\sigma_{m}$ is the ultimate stress of the reinforcement textile. Therefore, the textile warp perimeter and cross-sectional area related to the efficiency of reinforcement utilization were used for the calculation. Because of the same embedding length of the textile in the matrix, its effect on the bond stresses was not considered. The utilization factor β is defined as the warp circumference ratio to the reinforced material’s cross-sectional area. Both the warp circumference and the cross-sectional area were obtained from SEM.

Figure 11a,b show the PP and BFRP textile material utilization rates for the different numbers of layers (enhancement rate). The utilization rate of the PP textile decreased from 99.35% to 90.72%, while the enhancement rate increased from 1.3% to 4.82%. The PP textile utilization rate seemed insensitive to the enhancement rate, but the LECC textile reinforced by BFRP decreased from 84.11% to 65.21%, while the enhancement rate increased from 0.43% to 1.29%. From Equation (1), it can be seen that the enhancement rate has no effect on the bond strength; hence, when the utilization factor is the same, the material utilization decreases gradually as the number of layers increases. This is mainly because the textile is not fully utilized, and the utilization efficiency decreases as the number of layers increases. It was also found that the BFRP textile material utilization rate was lower when the utilization factor was significantly higher than that of the PP textile. This indicates that the material utilization rate was also related to the bonding performance of the material. Although increasing the perimeter of the warp improved the bond strength, due to the poor bonding performance, the material utilization was lower because the BFRP textile could not fully utilize the tensile stress. Figure 11c shows the material utilization rate of the BFRP grid in a one-layer arrangement; the BFRP grid had an enhancement rate from 1.3% to 4.21%, and its utilization rate decreased from 81.19% to 71.32%. It can be observed that the utilization rate decreased with the decrease in the utilization factor. The main reason for this phenomenon is that a larger utilization factor denoted a larger external perimeter of the yarn, which increased the contact area between the textile and the matrix, and the increased friction between the yarn and the matrix increased the bond strength. This means that the material utilization rate is not only controlled by the enhancement rate but is also related to the material utilization factor, which means that the ratio of the material’s circumference to the cross-sectional area positively affects the material utilization rate.

### 3.7. Effect of Enhancement Rate on LECC

**Figure 12:**
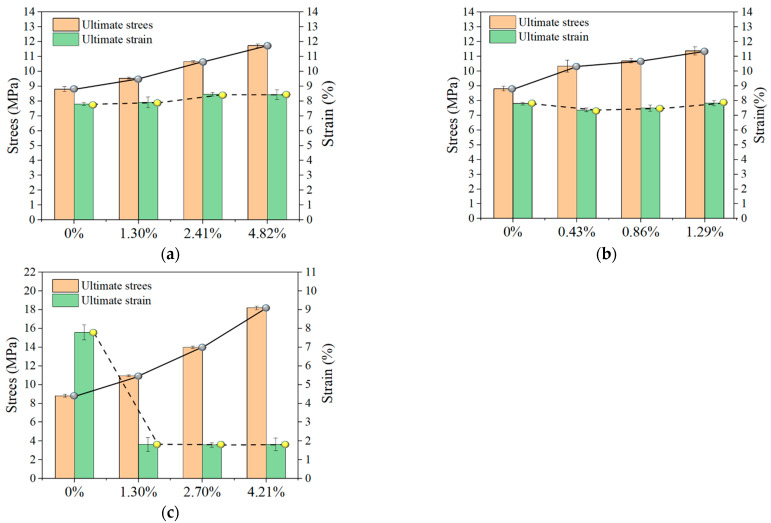
Comparison of ultimate stress and ultimate strain for different enhancement rates: (**a**) PP textile, (**b**) BFRP textile, and (**c**) BFRP grid.

In Section 3.5, it was noted that the arrangement form of the reinforcement material had a more negligible effect on the reinforcement effect of the LECC matrix. Thus, the effect caused by the change in the arrangement form was not considered in the TR-LECC. Moreover, on the basis of this conclusion, the effect of the reinforcement rate on the LECC matrix was analyzed again. Figure 12 shows the comparison diagram of the ultimate stress and ultimate strain under different reinforcement rates. With the increase in reinforcement rate, the ultimate stress of specimens was increased to a certain extent. The ultimate strain of the PP textile rose continuously with the addition of a reinforcement rate, while the ultimate strain of the BFRP textile and BFRP grid remained stable with the rise in the enhancement rate. It is not difficult to understand that the ultimate stress changes with the enhancement rate change, under the influence of many factors. For example, the ultimate strain of the reinforcing material plays a critical role. The PP textile-reinforced LECC exhibited a higher strain than the matrix due to its ultrahigh ductility, while the BFRP grid failed early due to the slight ultimate strain. In addition, the bond-slip effect can also increase the ultimate strain. The frictional force between the BFRP textile and the matrix after debonding and the bridging force of the PE fibers together maintain the ductility performance.

For the PP textile, increases in ultimate stress values of 8.3%, 21.1%, and 33.3% were achieved at the enhancement rates (1.30%, 2.41%, and 4.82%), while the ultimate strains increased by 1.5%, 8.5%, and 8.7%, respectively. On the other hand, the BFRP textile increased the ultimate stress by 17.5%, 21.6%, and 29.3% at its enhancement rates, and the ultimate strain was maintained by friction and the PE fiber bridging force as in the LECC matrix. The increases in ultimate stress for BFRP grids at the enhancement rate were 40.2%, 79.2%, and 133.5%, respectively, but the ultimate strain was maintained at only 1.8%. Combined with the study of Peled [57], it can be found that the strengthening effect is closely related to the textile’s material type, and the strengthening effect is more evident if the axial tensile strength is more significant. Meanwhile, the bonding performance of the textile material and the matrix is also a key factor because the tensile strength of the BFRP textile is about twice that of the BFRP grid. Nevertheless, the strengthening effect of the BFRP textile accounted for 83% of the BFRP grid; thus, the bonding performance also significantly influenced the strengthening effect.

## 4. Finite Element Simulation

**Figure 13:**
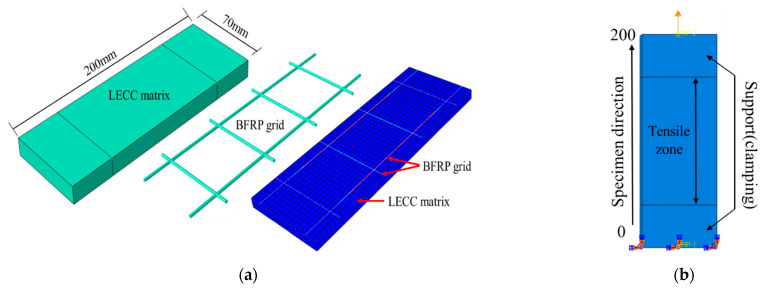
Finite element model. (**a**) Details of the LECC matrix and BFRP grid, (**b**) Schematic diagram of tensile test.

Accurate finite element simulation is a reliable analytical tool that not only simulates the overall response of structure but also enables the study of properties and effects that are difficult or impossible to determine experimentally, such as stress or strain distributions within the material. However, this is essential to understand the macroscopic structural behavior, which is based on the mechanical behavior at a much smaller scale. The authors of [26,58] provided a constitutive model of the ECC matrix and a constitutive relationship of the BFRP material. On the basis of these studies, the LECC matrix model used a C3D8R unit in the solid form. The BFRP material used a three-dimensional truss model (T3D2) for rod members that could only withstand tensile loads but not bending moments, as shown in Figure 13a.

### 4.1. Material Model

Since no relative slip was observed between the BFRP grid and the LECC matrix with perfective bonding properties, the interaction between the BFRP and the LECC matrix was simulated using the “embedded element” in ABAQUS. The interface effect between the LECC matrix and BFRP was achieved by defining “tensile stiffing” in the concrete damaged plasticity model. 

We established the reference point (RP) and the test clamping part to achieve the coupling through interaction and established a wholly fixed restraint method at the RP position; we continued to set the RP at the tensile end. Moreover, we continued to establish the coupling role of the clamping surface with the RP, and we finally completed the test using the displacement loading method, as shown in Figure 13b.

### 4.2. Results and Discussion

**Figure 14:**
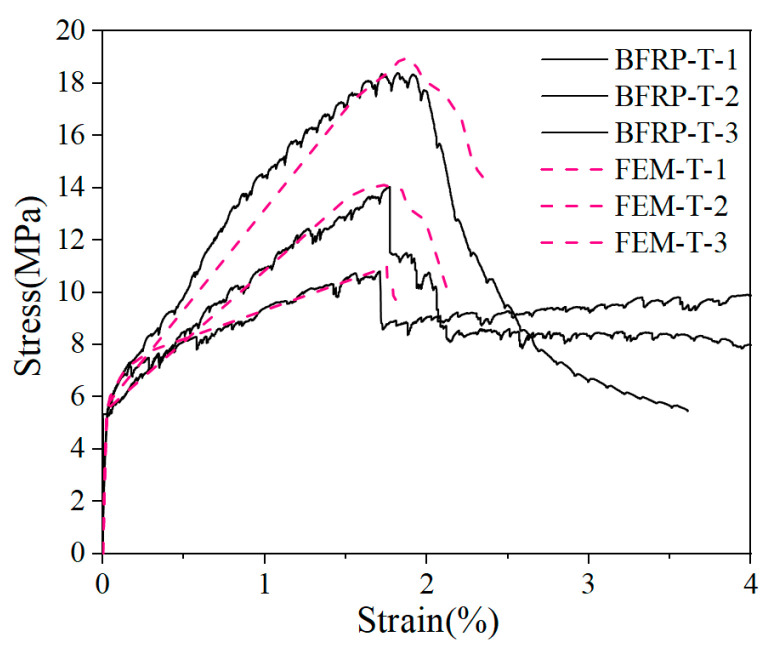
Stress–strain curves of the experimental results and numerical simulation results.

To validate the finite element model, the numerical model and the experimental results were compared in terms of both the tensile stress–strain curve and the failure mode of the specimen. The numerical behavior of the tensile stress–strain curves obtained from the finite element analysis with the experimental results is shown in Figure 14. The slope of the curve obtained from the finite element model was close to that of the experimental results; the elastic stage in the simulation almost coincided with that of the experimental curve, and the elastic modulus, peak stress, and ductility of the multi-slit cracking stage were highly consistent with the experimental results. This indicates that the modeling method can reflect the mechanical properties of the BFRP grid-reinforced LECC composite under axial tensile loading.

**Figure 15:**
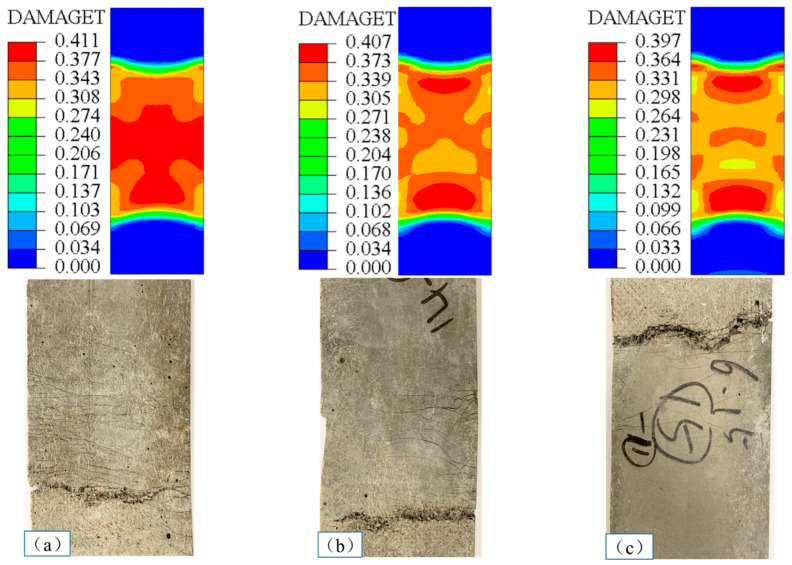
Failure modes for different enhancement rates in finite element simulations and tests: (**a**) 1.30%, (**b**) 2.70%, and (**c**) 4.21%.

The simulations revealed the tensile failure modes of the TR-LECC for different textile enhancement rates, as shown in Figure 15. It is noteworthy that the simulation test results for tensile damage distribution and crack development patterns were the same as the experimental results. As shown in Figure 15a, the middle region of the specimen suffered more tensile damage than other locations. It can be seen that there were more fine cracks in the central part of the specimen compared with the test, indicating that the tensile damage was also more significant in the central location in the test. Figure 15b,c show that the maximum tensile damage from the simulations was produced at the two ends, with relatively minor tensile damage in the middle region. Furthermore, the test specimens also had fewer cracks and significantly smaller crack widths in the middle before finally failing at the end of the tensile area specimen. From the finite element results of the three different reinforcement rates, it can be concluded that the tensile damage cracking conditions of the simulation and test are the same. The damage produced by the specimen during the tensile process was not severe, which explains why the number of cracks and crack width decreased in the test, indicating that the BFRP grid bore the main load in the tensile state, which not only fully shows the reinforcing effect and the inhibition of the development of cracks, but also shows the improvement to the durability performance of the TR-LECC composite.

### 4.3. The Impact of Arrangement Form

**Figure 16:**
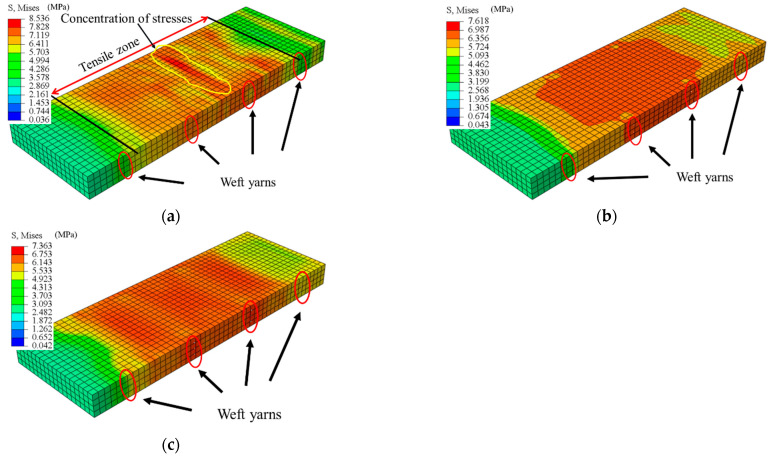
Stress distribution for different BFRP grid layers: (**a**) one-layer grid, (**b**) two-layer grid, and (**c**) three-layer grid.

Figure 16 shows the effects of three different arrangements on the stress distribution of the LECC matrix. When using a one-layer BFRP grid, the LECC matrix in the tensile region exhibited a nonuniform stress distribution, with a prominent stress concentration area in the middle of the model and gradually decreasing to the sides; the arrangement of the two-layer BFRP grid exhibited a better stress distribution, and the tensile region improved the problem of concentrated stress; the three-layer BFRP grid arrangement not only showed a uniform stress distribution in the whole tensile area but also a reduced stress value. From the finite element simulation, we can see that the internal BFRP grid arrangement mainly influenced the stress distribution of the LECC matrix. When the one-layer arrangement had a smaller bridging effect on the BFRP grid in the matrix, the stress concentration was formed after the matrix cracked. Finally, cracks gradually developed under a high-stress state and created major crack damage. However, the multi-layer grid arrangement provided a greater fiber-bridging effect. Thus, the tensile stress of the BFRP grid was uniformly transferred to the matrix material, avoiding the stress concentration in the reinforced material and improving the stress form of the matrix. In addition, the stress value of the matrix at the location of the weft yarn in the tensile area was significantly lower than that of the other parties, indicating that the presence of the weft yarn also changed the stress distribution and the magnitude of the stress value of the matrix.

**Figure 17:**
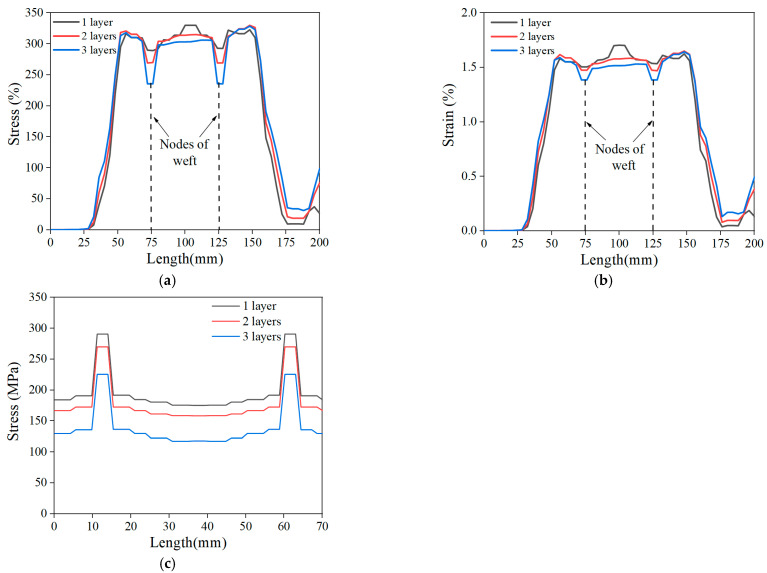
Stress–strain of BFRP grid: (**a**) stress in the length direction of the specimen, (**b**) strain in the length direction of the specimen, and (**c**) stress in the width direction of the specimen.

We further investigated the effect of the arrangement form on the stress and strain distribution; Figure 17 shows the stress and strain values along with the length and width directions of the specimen obtained for different layers of the BFRP grid. The more significant stress and strain values at the tensile end of the specimen were caused by setting the displacement loading surface of the load boundary. Then, the displacement loading caused tensile stress and strain in the tensile end section under tension. It can be seen that there was an apparent stress concentration in the middle of the specimen with a one-layer grid arrangement, and there was also a stress–strain mutation at the location of the weft yarn. Moreover, the stress in the specimen direction of the BFRP grid decreased gradually as the number of arrangement layers increased. Therefore, a multilayer arrangement of the grid can avoid the stress concentration problem and improve the stress distribution of the BFRP grid in the matrix, and the presence of the weft yarn can take up the stress in the warp direction.

### 4.4. Influence of Weft Yarn

**Figure 18:**
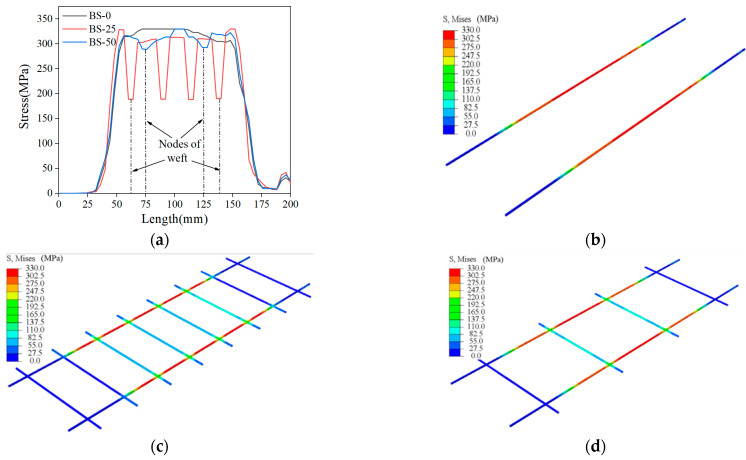
(**a**) Stress distribution pattern of weft yarn acting on the length direction of the specimen; (**b**) no weft yarn reinforcement, (**c**) grid weft yarn spacing of 25 mm, and (**d**) grid weft yarn spacing of 50 mm.

Figure 18a shows the stress distribution along the length of the specimen under tensile loading for three grids spacings, which contained no weft reinforcement (BS-0), a weft spacing of 25 mm (BS-25), and a weft spacing of 50 mm (BS-50), as shown in Figure 18b–d, respectively. It can be seen that the specimen without weft reinforcement reached the ultimate stress state at several locations, and the presence of weft reinforcement significantly reduced the ultimate stress and decreased the stress at the nodes of the weft and warp. With the reduction in the weft spacing, the stress concentration of the specimen was improved, which means that the presence of the weft not only helped the warp to bear part of the load but also improved the stress state by avoiding stress concentration. Due to the impregnation of the epoxy resin at the node, the weft and warp yarns formed a rigid node so that the weft yarns anchored to the matrix produced an interlocking effect on the warp yarns through the node. Finally, the interlocking effect could transfer the stress to the weft yarns through the node and help the warp yarns to bear part of the load, which was also confirmed by Lior [25] in his experiments.

## 5. Conclusions

On the basis of the LRS textile and LECCs, TR-LECCs with lightweight and high-ductility properties were developed in this research. Through experiments and FEM, the impacts of variables including textile type, enhancement rate, and arrangement form on the performance of TR-LECCs were investigated. According to the results of this work, the following findings can be summarized:

(1) It is possible to develop TR-LECCs with strengths similar to conventional TR-ECCs and with ultimate strains of 8.0% (3–4 times those of traditional TR-ECCs) by combining the LRS textile and LECCs. This can improve their significant energy dissipation capacity when used to strengthening and repair structures.
(2) Although the tensile characteristics of TR-LECCs are essentially unaffected by the type of textile arrangement (a multilayer arrangement or concentrated arrangement), TR-LECC cracking patterns are nevertheless impacted. While the concentrated arrangement greatly reduces the number of cracks and increases the crack width due to the stress concentration, the multilayer arrangement is advantageous for the fine dispersion of cracks.
(3) The textile type significantly influences the tensile performance of TR-LECCs. Because PP textiles have a higher tensile strain capacity (>8%), TR-LECC reinforcement provides much better strain ductility. Although this does not contribute as much to stiffness as traditional BFRP, increasing the enhancement rate can compensate for it. Due to bond-slip failures, BFRP textiles cannot fully utilize its reinforcing effect. However, BFRP grids impregnated with epoxy resin efficiently utilize the BFRP material’s reinforcing effect, increasing the LECC matrix’s tensile strength by 40.2% to 133.5%.
(4) The ultimate tensile stress of TR-LECCs improves significantly with an increasing enhancement rate of the textile, but the increase in the enhancement rate decreases the material utilization rate. For instance, as the enhancement rate increased from 0.43% to 1.29%, the material utilization of LECC reinforced with BFRP textile decreased from 84.11% to 65.21%. Notably, the utilization rate of PP textile seems insensitive to the enhancement rate, decreasing by just 8.63% as the enhancement rate increased from 1.3% to 4.82%, which is favorable for large-volume applications of PP textile.
(5) According to the FEM analysis, the arrangement forms considerably alter how the stress values are distributed in the TR-LECC. The centralized arrangement causes a stress concentration in the TR-LECC, and the stress value is large, while the multilayer arrangement facilitates uniform distribution of stress values in the TR-LECC. In addition, the presence of weft yarns has an important influence on the stress form in the TR-LECC; as the number of weft yarns increases, the stress concentration in the tensile region of the TR-LECC tends to weaken. On the other hand, adding additional weft yarns can help the warp yarns bear a higher axial load.

The current research demonstrates the feasibility of TR-LECC composites and provides an essential basis for the design of textile-reinforced LECC. However, more experiments and finite element simulations are needed to explore suitable reinforcing materials for concrete structure repair and strengthening and optimize contact interface properties for improvement.

## Figures and Tables

**Table 1:** The details of textile types.

Types	PP-1	PP-2	BFRP-F	BFRP-T-1	BFRP-T-2	BFRP-T-3
Textile size	30 mm	30 mm	25 mm	50 mm	50 mm	50 mm
Cross-section area	4.76 mm^2^	1.59 mm^2^	1.47 mm^2^	7.95 mm^2^	13.86 mm^2^	23.32 mm^2^

**Table 2:** The design of the TR-LECC specimens.

Specimen ID ^a^	Reinforcement Rate (%)	Material Types	Textile Grid Plies
PP-1-1.30	1.30	PP textile	1
PP-2-1.30	1.30	PP textile	2
PP-3-1.30	1.30	PP textile	3
PP-2-2.41	2.41	PP textile	2
PP-3-4.82	4.82	PP textile	3
BF-1-0.42	0.42	BFRP textile	1
BF-2-0.84	0.84	BFRP textile	2
BF-3-1.29	1.29	BFRP textile	3
BT-1-1.30	1.30	BFRP grid	1
BT-1-2.70	2.70	BFRP grid	1
BT-1-4.21	4.21	BFRP grid	1

Note: ^a^ Taking “PP-1-1.30” as an example, the first two letters are the type of the material in Table 2, the first number represents textile grid plies, and the second number denotes the reinforcement rate.

**Table 3:** Mechanical properties of LECC.

Cracking Stress (MPa)	Cracking Strain (%)	Ultimate Stress (MPa)	Ultimate Strain (%)
5.5 MPa	0.037%	8.8 MPa	7.8%

**Table 4:** Mix proportions of LECC mixtures (kg/m^3^).

Cement	Fly Ash	Nanosilica	Fly Ash Cenospheres	Water	Superplasticizer	PE Fiber
874	391.5	39	195.8	238.7	91.7	20

**Table 5:** Key mechanical property parameters of reinforced materials.

Material Types	Ultimate Tension (KN)	Young’s Elastic Modulus (GPa)	Ultimate Stress (MPa)	Ultimate Strain (%)
PP	0.646	-	102.17	12.14
BFRP-T	5.068	18.2	365.65	2.01
BFRP-F	1.061	25.5	719.56	2.82

## Data Availability

The data presented in this study are available from the corresponding author upon reasonable request.
